# Spathulenol, a Sesquiterpene from *Guarea macrophylla*, Displays Potent and Selective *Anthelmintic* Activity against *Angiostrongylus cantonensis*

**DOI:** 10.1021/acsomega.6c01587

**Published:** 2026-04-16

**Authors:** Juliana M. Santos, Lucas Fukui-Silva, Camila S. Amorim, Marina M. Gonçalves, João Pedro V. Moriconi, Igor S. Alborghetti, Roberto Baptista P. de Almeida, João Henrique G. Lago, Josué de Moraes

**Affiliations:** † Center for Research on Neglected Diseases, 92928 Guarulhos University, 07023-070 Guarulhos, São Paulo, Brazil; ‡ Center for Natural and Human Sciences, 74362 Federal University of the ABC, 09210-580 Santo Andre, São Paulo, Brazil; § Department of Botany, Institute of Biosciences, 99024 University of São Paulo, 05508-090 São Paulo, São Paulo, Brazil

## Abstract

Helminth infections remain among the most prevalent neglected tropical diseases worldwide, with angiostrongyliasis representing an emerging zoonotic condition associated with eosinophilic meningitis. In this study, spathulenol, a sesquiterpene isolated from *Guarea macrophylla* (Meliaceae), was evaluated for its anthelmintic potential using first-stage larvae (L1) of *Angiostrongylus cantonensis* as a phenotypic screening model. Spathulenol exhibited pronounced *in vitro* activity against L1 larvae, with an EC_50_ value in the low micromolar range (7.6 μM), outperforming the reference drug albendazole (15.1 μM) and showing slightly higher potency than pyrantel pamoate (10.4 μM) under identical experimental conditions. Phenotypic, morphological, and morphometric analyses further revealed compound-specific effects on parasite larvae, supporting biological differentiation from reference anthelmintics. This activity was accompanied by a favorable selectivity profile, as no cytotoxic effects were observed in human and animal cell lines at concentrations well above those required for antiparasitic activity (CC_50_ >200 μM). Moreover, spathulenol did not induce lethality in the free-living nematode *Caenorhabditis elegans* at concentrations up to 1000 μM. Taken together, these findings identify spathulenol as a selective hit compound emerging from phenotypic screening, representing a promising scaffold for early-stage anthelmintic drug discovery.

## Graphical abstract

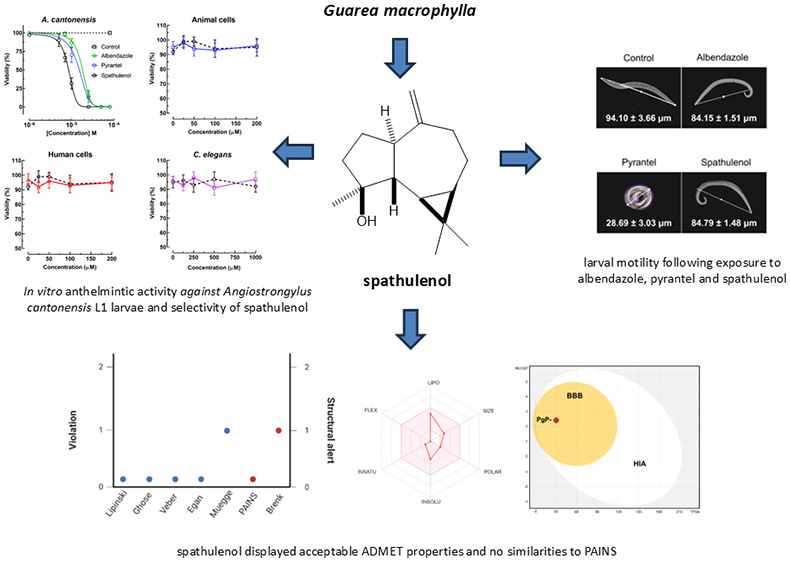

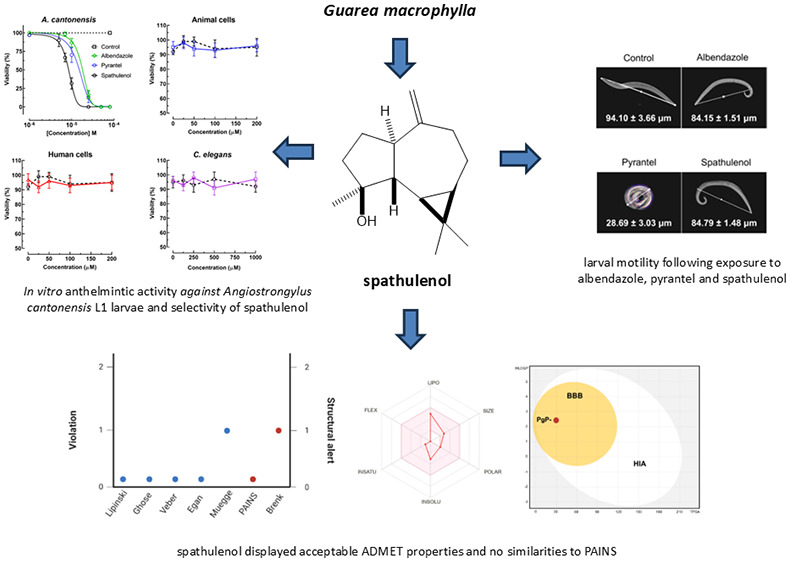

## 1 Introduction

Helminth infections remain among the most prevalent neglected tropical diseases worldwide, affecting more than one billion people and imposing a substantial burden on public health systems, particularly in low- and middle-income countries.^1^ These infections are strongly associated with poverty, inadequate sanitation, and limited access to healthcare, perpetuating cycles of social and economic vulnerability. Consequently, the control of helminthiases is closely aligned with several United Nations Sustainable Development Goals, including good health and well-being, clean water and sanitation, reduced inequalities, and sustainable communities.^2,3^

Despite their global impact, therapeutic options for helminth infections rely on a limited number of anthelmintic drugs that have been used for decades.^4^ This restricted pharmacological arsenal raises concerns regarding efficacy across different parasite species and developmental stages, as well as the potential emergence of drug resistance.^1,5^ In this context, the discovery of new anthelmintic candidates with improved selectivity and safety profiles remains a priority within the global agenda for neglected tropical diseases and drug development.^6,7^ More recently, *Angiostrongylus cantonensis*, commonly referred to as the rat lungworm, has attracted increasing attention not only as the etiological agent of angiostrongyliasis, a zoonotic infection associated with eosinophilic meningitis,^8–10^ but also as an experimentally tractable system for exploratory anthelmintic screening approaches.^11–13^ The use of noninfective larval stages, particularly first-stage larvae (L1), offers a practical and biologically relevant platform for phenotypic screening while minimizing biosafety concerns.^12,14^

Natural products have historically played a central role in the discovery of antiparasitic agents, and terpenoids represent one of the most structurally diverse classes of bioactive secondary metabolites.^15^ Among them, sesquiterpenes have attracted particular interest due to their relatively low molecular weight, favorable physicochemical properties, and recurrent biological activity across different experimental systems.^16^ Several sesquiterpenes and sesquiterpene-rich extracts have been reported to display antiparasitic, antimicrobial, and anti-inflammatory effects, often accompanied by low cytotoxicity in mammalian cells,^17–19^ reinforcing their relevance as starting points for early stage drug discovery.

Spathulenol is an oxygenated sesquiterpene commonly identified in the essential oils and extracts of various medicinal plants, including the Brazilian specie *Guarea macrophylla* (Meliaceae).^20^ As previously reported, this compound displays antimicrobial,^21^ insecticidal^22^ and anti-inflammatory^21,23^ activities in different experimental models, generally with a favorable preliminary safety profile. However, its anthelmintic potential has not yet been explored. In this context, the present study evaluated, for the first time, the *in vitro* activity of spathulenol isolated from leaves of *G. macrophylla* against L1 larvae of *A. cantonensis*, using albendazole and pyrantel pamoate as reference drugs. In parallel, *in vitro* cytotoxicity was evaluated against human and animal cell lines, while *in vivo* toxicity was assessed using the *Caenorhabditis elegans* model. Furthermore, *in silico* analyses were conducted to characterize the physicochemical and pharmacokinetic properties. Together, these approaches aim to position spathulenol as a selective hit compound within a phenotypic screening strategy for anthelmintic drug discovery.

## 2 Materials and Methods

### 2.1 General Procedures

Silica gel (Merck, 230–400 mesh) was used for column chromatographic separation procedures, while silica gel 60 PF_254_ (Merck) was used for analytical TLC (0.25 mm). Other reagents were purchased from Sigma-Aldrich (Brazil). NMR spectra were recorded on a Bruker Ascend Evo 600 spectrometer, operating at 600 and 150 MHz to the ^1^H and ^13^C nuclei, respectively. CDCl_3_ (Aldrich) was used as the solvent and TMS (Aldrich) was used as the internal standard. Chemical shifts (δ) are reported in ppm and the coupling constant (*J*) in Hz. GC-MS analysis was performed in a Shimadzu GC-17A chromatograph using an RtX-5 capillary column (5% phenyl, 95% poly­(dimethylsiloxane), 30 m × 0.25 mm × 0.25 μm film thickness; Restek) interfaced with an MS-QP-2010 and quadrupole mass analyzer with electron ionization (EI), operating at 70 eV.

### 2.2 Plant Material

*G. macrophylla* Vahl. ssp. Tuberculata (Meliaceae) leaves (1.2 kg) were collected at University of Sao Paulo campus, Sao Paulo city, SP, Brazil, in October 2025 and were identified by MSc. Roberto Baptista Pereira de Almeida. Voucher specimen (R.B. Almeida 1521) has been deposited at the Herbarium of Institute of Biosciences from University of São Paulo (IB-USP), São Paulo, SP, Brazil.

### 2.3 Extraction and Isolation

The dried and powdered leaves of *G. macrophylla* (400 g) were extracted with hexane (6 × 1 L) at room temperature to afford 10.4 g of crude extract after evaporation of the solvent under reduced pressure. Part of crude hexane extract (10.0 g) was chromatographed over a silica gel column (400 g, 45 cm × 2.5 cm) eluted with increasing amounts of EtOAc in hexane (9:1, 8:2, 7:3, 1:1, 3:7, 2:8, and 1:9) to afford 57 fractions (75 mL each) which were pooled together in seven groups (A–G) after TLC analysis. Fraction B (778 mg) was chromatographed over a silica gel column (150 g, 30 cm × 2 cm) eluted with hexane:EtOAc 8:2 to give 72 mg of spathulenol.

*Spathulenol*. Pale yellow oil. ^1^H NMR (600 MHz, CDCl_3_) δ/ppm: 4.67 (s, H-15b), 4.64 (s, H-15a), 2.39 (td, *J* = 14.1 and 6.3 Hz, H-1), 1.27 (s, H-14), 1.04 (s, H-13), 1.03 (s, H- 12), 0.70 (dd, *J* = 11.2, 9.5, and 6.1 Hz), 0.45 (dd, *J* = 11.2 and 9.5 Hz, H-6). ^13^C NMR (150 MHz, CDCl_3_) δ/ppm: 153.5 (C-10), 106.4 (C-15), 81.0 (C-4), 54.4 (C-1), 53.5 (C-5), 41.8 (C-3), 39.0 (C-9), 30.0 (C-6), 28.8 (C-12), 27.6 (C-7), 26.8 (C-2), 26.1 (C-14), 24.9 (C-8), 20.3 (C-11), 16.4 (C-13). EI-MS (70 eV) *m*/*z* (rel. int.): 220 (2), 205 (81), 202 (12), 187 (42), 177 (19), 159 (84), 147 (61), 119 (100), 105 (80), 91 (93), 43 (97).

### 2.4 Parasite Maintenance, Ethical Approval, and Larval Stages

The life cycle of *A. cantonensis* was maintained under laboratory conditions at the Research Center on Neglected Diseases, Guarulhos University, using procedures routinely established by our group. The parasite strain employed in this study was the NPDN-AC strain. Definitive hosts (*Rattus norvegicus*, Wistar rats) and intermediate hosts (*Biomphalaria glabrata* snails) were housed under controlled environmental conditions with ad libitum access to food and water.^11^ All experimental procedures involving animals were conducted in strict accordance with national and institutional guidelines for the care and use of laboratory animals and were approved by the Institutional Animal Care and Use Committee (IACUC) of Guarulhos University (approval number: 64/2024). First-stage larvae (L1) of *A. cantonensis* were obtained from fecal samples of infected *R. norvegicus* using standard sedimentation procedures.^24^ Larvae were washed three times in RPMI 1640 medium supplemented with penicillin (100 U/mL) and streptomycin (100 μg/mL) prior to use. Only active and morphologically intact larvae were selected for *in vitro* assays. L1 larvae were chosen as a noninfective developmental stage suitable for phenotypic screening and anthelmintic drug discovery.

### 2.5 *In Vitro* Anthelmintic Assay

The anthelmintic activity of spathulenol was evaluated against L1 larvae of *A. cantonensis* using a motility and viability-based assay. Approximately 50 larvae were allocated per well in 96-well plates containing RPMI 1640 medium in a final volume of 200 μL.^25^ Spathulenol was tested at increasing concentrations prepared from stock solutions in dimethyl sulfoxide (DMSO), with a maximum final DMSO concentration of 0.5% (v/v) in all wells. Albendazole and pyrantel pamoate were included as a reference drug under the same experimental conditions. Control groups consisted of larvae incubated with medium containing 0.5% DMSO only. Larval motility and viability were monitored by optical microscopy after incubation for predetermined time points. Larvae were considered nonviable when complete loss of motility was observed, even after mechanical stimulation.^26^ Concentration–response curves were generated, and half-maximal effective concentrations (EC_50_) were calculated by nonlinear regression analysis.^27^ All experiments were performed in three independent biological replicates, each conducted in triplicate.

### 2.6 Morphological and Morphometric Analysis of *A. cantonensis* L1 Larvae

Morphological alterations in *A. cantonensis* L1 larvae following exposure to spathulenol, albendazole, or pyrantel pamoate were evaluated by optical microscopy. After incubation under the same conditions described in the anthelmintic assay, larvae were examined using an inverted microscope (MOTIC AE2000, Canada). Representative images were captured using the microscope-integrated imaging system.

For morphometric analysis, larval body length was measured from captured images using the proprietary image analysis software associated with the microscope (Motic Images Plus 3.0). At least six larvae per experimental group were randomly selected and measured in each independent experiment. Data were expressed as mean ± standard deviation, and statistical comparisons between treated and control groups were performed as described in the Statistical Analysis section.

### 2.7 Cytotoxicity Assays in Mammalian Cell Lines

The cytotoxicity of spathulenol was evaluated in human keratinocyte (HaCaT) and African green monkey kidney epithelial (Vero) cell lines. Cells were maintained under standard culture conditions and seeded into 96-well plates.^28^ After overnight attachment, cells were exposed to compounds at an increasing concentration for 24 h. The maximum concentration tested was 200 μM, with DMSO at a final concentration not exceeding 0.5% (v/v). Cell viability was determined using a colorimetric viability assay according to established protocols routinely applied by our group.^29^ Cells treated with culture medium containing 0.5% DMSO served as vehicle controls. Cytotoxicity data were expressed as percentage of viable cells relative to control wells.^30^ Experiments were performed in three independent assays, each in triplicate.

### 2.8 Toxicity Assessment in *Caenorhabditis elegans*

To further assess selectivity, the toxicity of spathulenol was evaluated in the free-living nematode *Caenorhabditis elegans* (Bristol N2 strain).^31^ Worms were maintained on nematode growth medium (NGM) agar plates seeded with *Escherichia coli* OP50 under standard laboratory conditions.^32^ Synchronized ions were exposed to compounds at concentrations up to 1000 μM in liquid medium, with a maximum final DMSO concentration of 0.5% (v/v). Worm viability and motility were evaluated microscopically after exposure. Worms showing complete immobility and lack of response to mechanical stimulation were considered nonviable.^33^ All assays were conducted in three independent experiments performed in triplicate.

### 2.9 *In Silico* ADME Analysis

*In silico* prediction of physicochemical and pharmacokinetic properties of spathulenol was performed using the SwissADME web-based platform.^34^ Parameters related to molecular weight, lipophilicity, polarity, solubility, gastrointestinal absorption, and drug-likeness were calculated using default settings. These analyses were conducted to provide a preliminary assessment of the compound’s ADME-related properties within an early stage drug discovery context.

### 2.10 Statistical Analysis

Concentration–response data and half-maximal effective concentrations (EC_50_) are expressed as mean ± standard deviation (SD) and were obtained by nonlinear regression analysis using GraphPad Prism 8.0 software. Morphometric data are expressed as mean ± standard error of the mean (SEM) and were analyzed by one-way analysis of variance (ANOVA), followed by Tukey’s post hoc test for multiple comparisons. All experiments were conducted as three independent biological replicates, each performed in technical triplicate. Differences were considered statistically significant when *P* < 0.05.

## 3 Results

### 3.1 Structural Identification of Spathulenol

The leaves of *G. macrophylla* yielded a sesquiterpene (99% of purity by GC), identified through analysis of its MS and NMR data as aromadendr-10(15)-en-4β-ol,^35^ commonly referred to as spathulenol (Figure 1).

**Figure 1:**
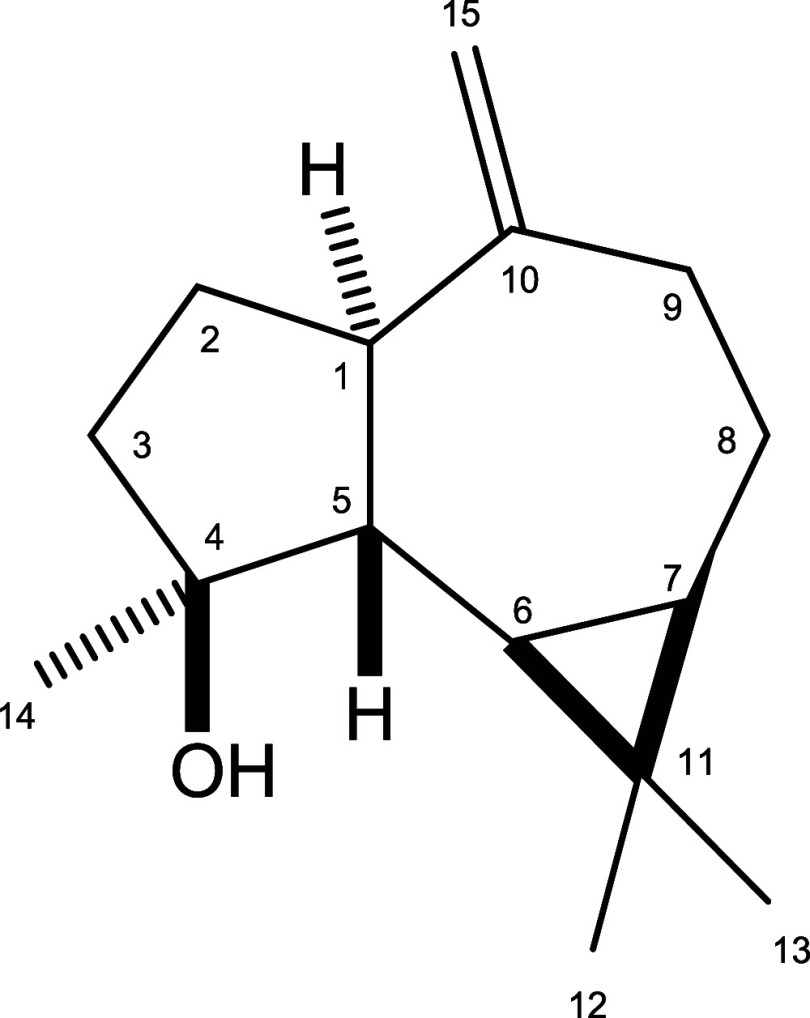
Chemical structure of the sesquiterpene spathulenol isolated from the leaves of *G. macrophylla* (Meliaceae).

### 3.2 *In Vitro* Anthelmintic Activity against *A. cantonensis* L1 Larvae

Spathulenol exhibited pronounced *in vitro* anthelmintic activity against first-stage larvae (L1) of *A. cantonensis* in a concentration-dependent manner, with an EC_50_ value of 7.6 μM, indicating effective larval inhibition at low micromolar concentrations. Under the same experimental conditions, the reference drugs albendazole and pyrantel pamoate displayed EC_50_ values of approximately 15 μM and 10 μM, respectively (Table 1). Statistical analysis demonstrated that spathulenol was significantly more potent than albendazole (*P* < 0.001) and pyrantel pamoate (*P* < 0.05) in this model.

**Table 1:** *In Vitro* Anthelmintic Activity and Selectivity Profile of Spathulenol Compared with Albendazole and Pyrantel Pamoate^a^,^b^,^c^,^d^

compound	*A. cantonensis* L1 EC_50_ (μM)	animal cells CC_50_ (μM)	human cells CC_50_ (μM)	SI^e^	*C. elegans* LD_50_ (μM)
Spathulenol	7.6 ± 0.9^f^	>200	>200	>26	>1000
Albendazole	15.1 ± 1.2	>200	>200	>13	16.2 ± 1.4
Pyrantel pamoate	10.4 ± 0.6	>200	>200	>19	25.3 ± 3.8

a Anthelmintic activity was evaluated against first-stage larvae (L1) of *A. cantonensis*. Cytotoxicity was assessed in mammalian Vero (animal) and HaCaT (human) cell lines. Toxicity toward nonparasitic nematodes was evaluated using *C. elegans*. Data are expressed as mean ± standard deviation from three independent experiments performed in triplicate.

b EC_50_: concentration required to reduce larval motility/viability by 50%.

c CC_50_: concentration causing 50% reduction in cell viability.

d LD_50_: concentration causing 50% lethality in *C. elegans*.

e SI (Selectivity Index) = CC_50_/EC_50_.

f *P* < 0.001 compared with albendazole.

Dose–response curves for all compounds are shown in Figure 2, illustrating the comparative larvicidal profiles of spathulenol, albendazole, and pyrantel pamoate across the tested concentration range.

**Figure 2:**
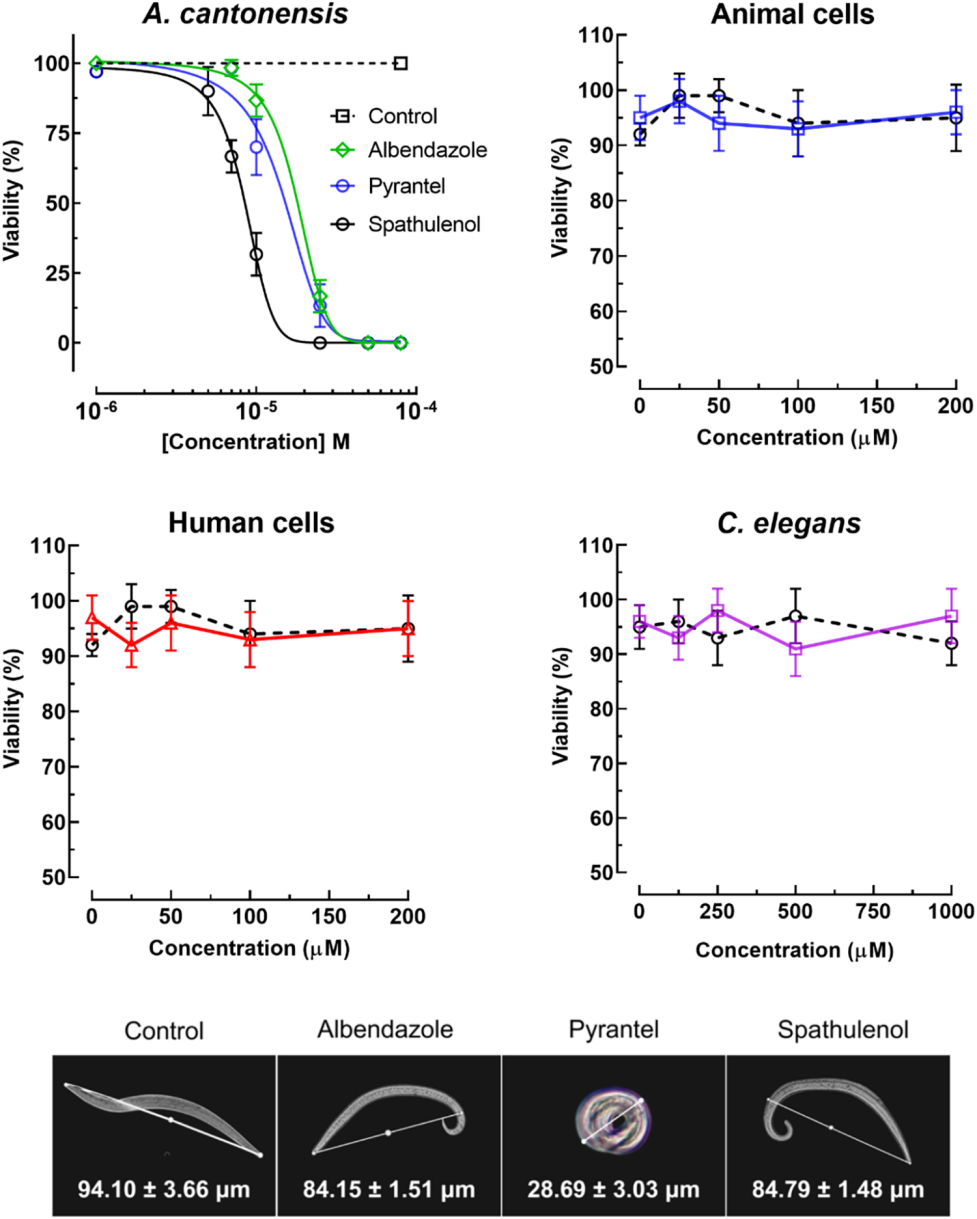
*In vitro* anthelmintic activity, selectivity, and phenotypic effects of spathulenol against *A. cantonensis* L1 larvae. Concentration–response curves illustrating the *in vitro* anthelmintic activity of spathulenol and the reference drugs albendazole and pyrantel pamoate against first-stage larvae (L1) of *A. cantonensis*, assessed based on loss of larval motility following exposure to compounds. Selectivity was evaluated by determining the viability of mammalian Vero (animal) and HaCaT (human) cell lines after 24 h of exposure to spathulenol, as well as by toxicity assessment in the free-living nematode *C. elegans*. Representative light microscopy images and corresponding morphometric analysis show compound-specific morphological effects on *A. cantonensis* L1 larvae following treatment with spathulenol, albendazole, or pyrantel pamoate. Data points represent mean ± standard deviation from three independent experiments performed in triplicate. Concentration–response curves were generated by nonlinear regression analysis to determine EC_50_ values.

### 3.3 Cytotoxicity Assessment in Mammalian Cell Lines

The cytotoxicity of spathulenol was evaluated in mammalian Vero (animal) and HaCaT (human) cell lines. No detectable cytotoxic effects were observed for spathulenol at the highest concentration tested (200 μM) in either cell line. Similarly, albendazole and pyrantel pamoate did not induce cytotoxicity in Vero or HaCaT cells up to 200 μM (Figure 2). Based on these results, the selectivity index (SI), calculated as the ratio between CC_50_ and EC_50_ values, was greater than 26 for spathulenol, greater than 13 for albendazole, and greater than 19 for pyrantel pamoate (Table 1). These findings indicate a favorable selectivity profile for spathulenol, with antiparasitic activity occurring at concentrations substantially lower than those associated with cytotoxicity in mammalian cells.

### 3.4 Toxicity Evaluation in *Caenorhabditis elegans*

To further evaluate selectivity toward parasitic nematodes, spathulenol toxicity was assessed using the free-living nematode *C. elegans*, a widely used nonparasitic model for toxicity and safety evaluation. Spathulenol did not induce lethality or overt toxicity in *C. elegans* at concentrations up to 1000 μM (Figure 2), indicating a wide margin between antiparasitic activity against A. cantonensis and toxicity in a nonparasitic nematode model.

In contrast, both reference drugs exhibited measurable toxicity in *C. elegans*, with albendazole and pyrantel pamoate displaying LD_50_ values of approximately 16 μM and 25 μM, respectively (Table 1). These results further highlight the differential biological effects of spathulenol and reference anthelmintics across nematode species and support the selective antiparasitic profile of spathulenol.

### 3.5 Morphological and Morphometric Analysis of *A. cantonensis* L1 Larvae

Morphological alterations in *A. cantonensis* L1 larvae were evaluated by optical microscopy following exposure to spathulenol, albendazole, or pyrantel pamoate. Representative images revealed distinct phenotypic effects depending on the compound tested (Figure 2). Larvae exposed to spathulenol or albendazole largely retained an elongated morphology, whereas pyrantel pamoate treatment induced marked larval coiling and body contraction.

Quantitative morphometric analysis corroborated these observations. Control larvae exhibited an average length of approximately 94 μm, while larvae treated with spathulenol or albendazole showed moderate reductions in length, measuring approximately 85 μm. In contrast, pyrantel-treated larvae displayed a pronounced reduction in length, averaging approximately 29 μm, which was statistically significant when compared with control, spathulenol-, and albendazole-treated groups (*P* < 0.01).

### 3.6 *In Silico* ADME and Drug-likeness Profiling

*In silico* analyses performed using the SwissADME platform indicated that spathulenol presents physicochemical properties compatible with early stage small-molecule drug discovery. The compound complied with major pharmaceutical drug-likeness filters, including Lipinski, Ghose, Veber, and Egan criteria, with no violations detected. A single violation was observed under the Muegge filter, related to the low number of heteroatoms (Figure 3).

**Figure 3:**
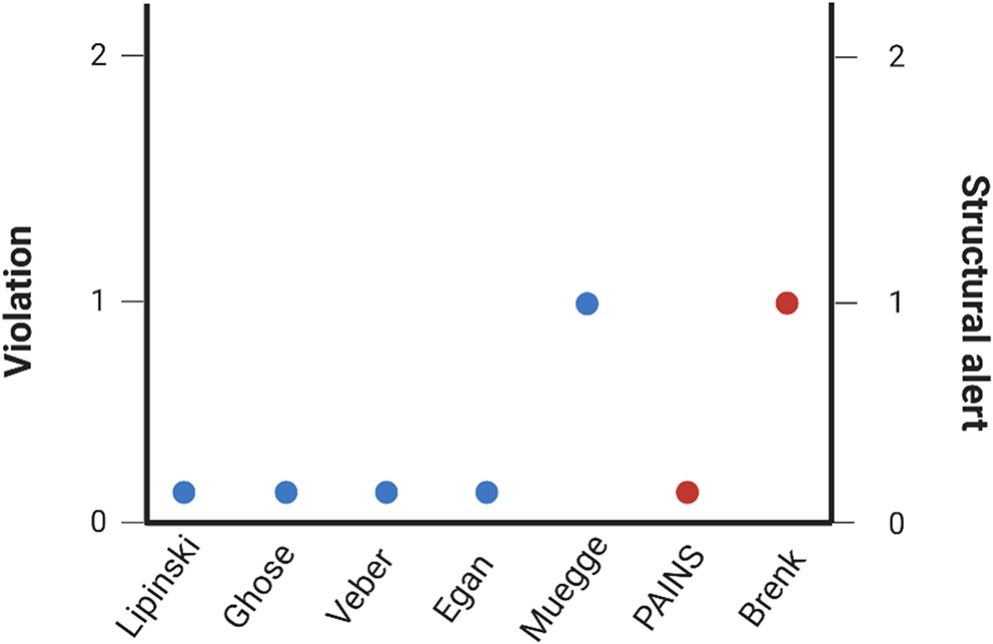
Drug-likeness and medicinal chemistry assessment of spathulenol. Compliance of spathulenol with major pharmaceutical drug-likeness filters, including Lipinski (Pfizer), Ghose (Amgen), Veber (GSK), Egan (Pharmacia), and Muegge (Bayer) criteria. Spathulenol complies with Lipinski, Ghose, Veber, and Egan rules, presenting a single violation under the Muegge filter related to the low number of heteroatoms. Structural alert analysis based on PAINS (pan-assay interference compounds) and Brenk filters revealed no PAINS alerts and one Brenk alert associated with an isolated alkene moiety. Predictions were generated using the SwissADME platform.

Physicochemical profiling showed that spathulenol has a molecular weight of 220.35 Da, low polarity (TPSA = 20.23 Å^2^), a high fraction of sp^3^ carbons (fraction Csp^3^ = 0.87), no rotatable bonds, and moderate lipophilicity, with a consensus log *P* value of 3.30. These parameters fall within reference ranges commonly associated with favorable oral bioavailability, as illustrated by the bioavailability radar (Figure 4). Pharmacokinetic predictions suggested high passive gastrointestinal absorption and blood–brain barrier permeability, as indicated by the BOILED-Egg model (Figure 4). Spathulenol was classified as a nonsubstrate of P-glycoprotein, suggesting a low likelihood of active efflux.

**Figure 4:**
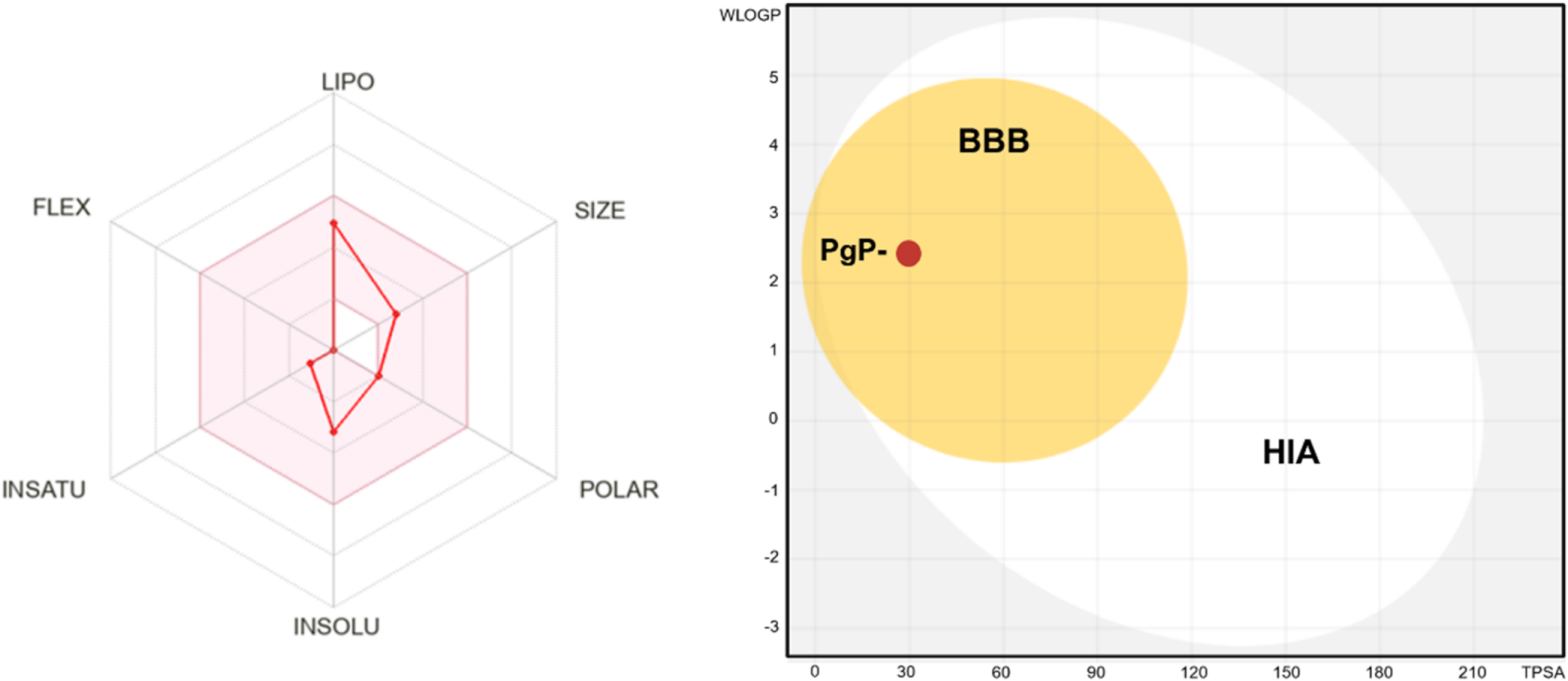
*In silico* pharmacokinetic profiling of spathulenol. Left panel: bioavailability radar depicting key physicochemical properties, including lipophilicity, molecular size, polarity, solubility, flexibility, and saturation, plotted within reference ranges associated with favorable oral bioavailability. Right panel: prediction of passive gastrointestinal absorption (HIA) and blood–brain barrier (BBB) permeability based on the BOILED-Egg model. The white region represents a high probability of passive gastrointestinal absorption, whereas the yellow region (yolk) indicates high BBB penetration potential. The red marker denotes spathulenol classification as a nonsubstrate of P-glycoprotein. Predictions were generated using the SwissADME platform.

Medicinal chemistry filters revealed no PAINS alerts and a single Brenk alert associated with an isolated alkene moiety (Figure 3). Together, these in silico data provide a complementary assessment of the physicochemical and pharmacokinetic profile of spathulenol within a phenotypic screening framework for anthelmintic drug discovery.

## 4 Discussion

The present study demonstrates that spathulenol, a sesquiterpene isolated from the leaves of *G. macrophylla*, exhibits pronounced *in vitro* anthelmintic activity against first-stage larvae of *A. cantonensis*, combined with a favorable selectivity profile across mammalian cell lines and a free-living nematode model. Beyond its relevance as an experimental system, *A. cantonensis* is a clinically important zoonotic parasite and the etiological agent of angiostrongyliasis, a helminthiasis associated with eosinophilic meningitis and an expanding geographic distribution.^36,37^ In this context, the identification of bioactive compounds against *A. cantonensis* remains of direct interest both for advancing therapeutic research on angiostrongyliasis and for expanding the repertoire of chemical scaffolds with anthelmintic potential.

Importantly, this work also highlights the value of biodiversity-driven drug discovery. Spathulenol is a plant-derived secondary metabolite for which no previous antiparasitic activity against helminths or protozoa has been reported. Its identification as an active compound against *A. cantonensis* therefore represents a novel biological property, reinforcing the role of natural products as a rich and still underexplored source of chemically diverse molecules for anthelmintic discovery. The isolation of an active sesquiterpene from *G. macrophylla* further underscores the importance of investigating plant biodiversity as a strategy to uncover new bioactive scaffolds relevant to neglected parasitic diseases.^15^

Spathulenol displayed an EC_50_ value in the low micromolar range against *A. cantonensis* L1 larvae, outperforming albendazole and showing slightly higher potency than pyrantel pamoate under the same experimental conditions. While albendazole and pyrantel pamoate remain important anthelmintics in clinical practice, both exhibit well-documented limitations related to spectrum of activity, stage specificity, and tolerability.^38,39^ In this context, the higher *in vitro* potency of spathulenol should not be interpreted as an indication of superior therapeutic efficacy, but rather as evidence of intrinsic antiparasitic activity that warrants further investigation within an early stage drug discovery framework. Notably, the absence of cytotoxicity in HaCaT and Vero cells at concentrations far exceeding the antiparasitic EC_50_ values, together with the lack of toxicity in *C. elegans*, indicates a wide selectivity window.

The use of *C. elegans* as a nonparasitic nematode model provided an additional layer of selectivity assessment.^40,41^ In contrast to spathulenol, both albendazole and pyrantel pamoate exhibited measurable toxicity in this organism, consistent with their known biological targets.^42^ This divergence highlights species and target-dependent effects among nematodes and reinforces the value of combining parasitic and free-living nematode models during early stage screening.^43^ Similar selectivity patterns have been reported by our group for other natural products and synthetic derivatives evaluated against *A. cantonensis*, including piplartine,^44^ cubebin,^14^ and 1,10-phenanthroline-5,6-dione based compounds,^12^ where antiparasitic activity was consistently observed at concentrations well below those associated with toxicity in nontarget systems.

Beyond differences in potency, the distinct morphological and morphometric effects observed among the tested compounds provide additional insight into their biological actions. Pyrantel pamoate induced a characteristic coiled and contracted phenotype in *A. cantonensis* L1 larvae, accompanied by a pronounced reduction in larval length. This phenotype is consistent with the established mechanism of action of pyrantel pamoate as a depolarizing neuromuscular agent acting on nicotinic acetylcholine receptors, leading to sustained muscle contraction and paralysis.^45,46^ In contrast, larvae exposed to spathulenol or albendazole largely retained an elongated morphology, despite reduced motility and viability, and exhibited only moderate reductions in body length. These observations indicate that spathulenol does not share the neuromuscular mechanism associated with pyrantel pamoate and instead likely operates through a distinct biological pathway.

While these phenotypic signatures support mechanistic differentiation, they are not sufficient to define specific molecular targets. Nevertheless, based on its physicochemical profile and previously reported biological activities of sesquiterpenes, some plausible hypotheses can be considered. Due to its lipophilic nature, spathulenol may interact with parasite membranes, potentially affecting membrane integrity or permeability.^47^ In addition, sesquiterpenes have been associated with mitochondrial dysfunction and disruption of energy metabolism in different biological systems,^48,49^ suggesting that similar mechanisms may contribute to the observed loss of viability in *A. cantonensis* larvae. These hypotheses remain speculative and warrant further investigation through dedicated mechanistic studies.

Within a broader comparative context, the activity of spathulenol against *A. cantonensis* L1 larvae can be considered alongside previous studies involving structurally diverse natural products and synthetic compounds. Piplartine, an amide isolated from *Piper truncatum*,^44^ and cubebin, a lignan obtained from *Drimys andina*,^14^ both exhibited potent antiparasitic activity against *A. cantonensis* larvae while maintaining favorable selectivity profiles. In parallel, chemically diverse synthetic molecules and rationally designed derivatives have also been shown to display reproducible and selective activity in this experimental system.^11,12,25^ Collectively, these findings indicate that antiparasitic effects observed in *A. cantonensis* larvae are not restricted to a specific chemical class or natural-product scaffold, supporting its use as an exploratory platform for the identification of chemically diverse anthelmintic hits.

From a chemical and biological perspective, spathulenol belongs to a class of sesquiterpenes widely distributed in essential oils and plant extracts, for which a broad spectrum of biological activities has been reported, including antimicrobial,^21^ insecticidal,^22^ and anti-inflammatory effects,^21,23^ often in the absence of marked cytotoxicity. This combination of structural diversity, bioactivity, and low cytotoxicity highlights the relevance of sesquiterpenes as chemically diverse and biologically tractable scaffolds for antiparasitic drug discovery. In addition, several sesquiterpenes have been reported to display anthelmintic activity in different helminth models, including compounds such as nerolidol,^50^ cnicin,^51^ budlein-A^52^ 3,6-epidioxy-bisabola-1,10-diene,^53^ polygodial,^54^ estafietin,^55^ and artemisinin-derived structures,^56^ further supporting the notion that this class of natural products represents a relevant chemical space for antiparasitic drug discovery. Although the present work does not address the molecular mechanism underlying its anthelmintic activity, the consistent and reproducible effects observed against *A. cantonensis* larvae support a direct effect on nematode viability. Further studies will be required to elucidate the molecular targets involved and to determine whether similar effects can be observed in other parasitic nematodes.

In this context, the *in silico* ADME analysis provides additional insight into the relationship between the physicochemical properties of spathulenol and its observed biological activity. The relatively low polarity (TPSA) and moderate lipophilicity of spathulenol are consistent with favorable passive membrane permeability, which may facilitate compound access to intracellular targets in nematodes. The predicted high gastrointestinal absorption further supports its potential as an orally bioavailable scaffold. Importantly, the combination of membrane permeability, predicted oral absorption, and lack of cytotoxicity is consistent with the selective antiparasitic profile observed *in vitro*. The absence of PAINS alerts and compliance with major drug-likeness filters further reinforce its suitability as a chemically tractable starting point for further optimization. In addition, the predicted ability of spathulenol to cross the BBB may be particularly relevant in the context of angiostrongyliasis, a disease characterized by central nervous system involvement. While BBB permeability could represent a desirable feature for targeting parasites in neural tissues, its pharmacological relevance remains to be experimentally validated in future *in vivo* studies.

Despite these promising findings, several limitations should be acknowledged. The biological evaluations were restricted to *in vitro* models, and the activity of spathulenol against other parasitic nematodes or additional developmental stages remains to be determined. Moreover, the in silico ADME and drug-likeness predictions provide only preliminary estimates of physicochemical and pharmacokinetic properties and require experimental validation through dedicated absorption, distribution, metabolism, and toxicity studies. As a natural product, spathulenol may also present challenges related to bioavailability, metabolic stability, and scalability, which would need to be addressed through structural optimization or formulation strategies. These limitations are typical of early stage natural-product hits and do not detract from their value as starting points for anthelmintic drug discovery.

In summary, this study identifies spathulenol as a selective and potent *in vitro* hit compound emerging from phenotypic screening, representing a promising scaffold for early stage anthelmintic drug discovery rather than a fully validated lead candidate. By integrating phenotypic screening in a parasitic nematode with selectivity assessment in mammalian cells and a free-living nematode model, this work contributes to the expanding landscape of exploratory strategies aimed at identifying chemically diverse candidates for neglected parasitic diseases. Further investigations, including structure–activity relationship studies, mechanistic characterization, and *in vivo* validation, will be essential to advance spathulenol along the drug discovery pipeline and to define its translational potential.

## Supplementary Material



## Data Availability

The data that support the findings of this study are available throughout the manuscript and supporting files.

## Notes

^1^H NMR spectrum of spathulenol (δ, 600 MHz, CDCl_3_) (Figure S1); ^13^C NMR spectrum of spathulenol (δ, 150 MHz, CDCl_3_) (Figure S2); GC/MS analysis (EI, 70 eV) of spathulenol (Figure S3) (PDF)
