# Supplementary material for: Spathulenol, a Sesquiterpene from Guarea macrophylla, Displays Potent and Selective Anthelmintic Activity against Angiostrongylus cantonensis
Source: ACS Omega. 2026 Apr 16;11(16):24770–8. doi: 10.1021/acsomega.6c01587 (PMC13129838; doi:10.1021/acsomega.6c01587)
Supplement: Supplementary file 1 [file ao6c01587_si_001.pdf]

Spathulenol, a sesquiterpene from *Guarea macrophylla*, displays potent and selective *anthelmintic* activity against *Angiostrongylus cantonensis*

Juliana M. Santos[1], Lucas Fukui-Silva[1], Camila S. Amorim[1], Marina M. Gonçalves[2], João Pedro V. Moriconi[2], Igor S. Alborghetti[2], Roberto Baptista P. de Almeida[3], João Henrique G. Lago[2,*], and Josué de Moraes[1,*]

[1]Center for Research on Neglected Diseases, Guarulhos University, 07023-070, Guarulhos, SP, Brazil

[2]Center for Natural and Human Sciences, Federal University of the ABC, 09210-580, Santo Andre, SP, Brazil

[3]Department of Botany, Institute of Biosciences, University of São Paulo, 05508-090, São Paulo, SP, Brazil.

SUPPORTING INFORMATION

*Correspondence: João Henrique G. Lago (joao.lago@ufabc.edu.br) and Josué de Moraes (moraesnpdn@gmail.com)

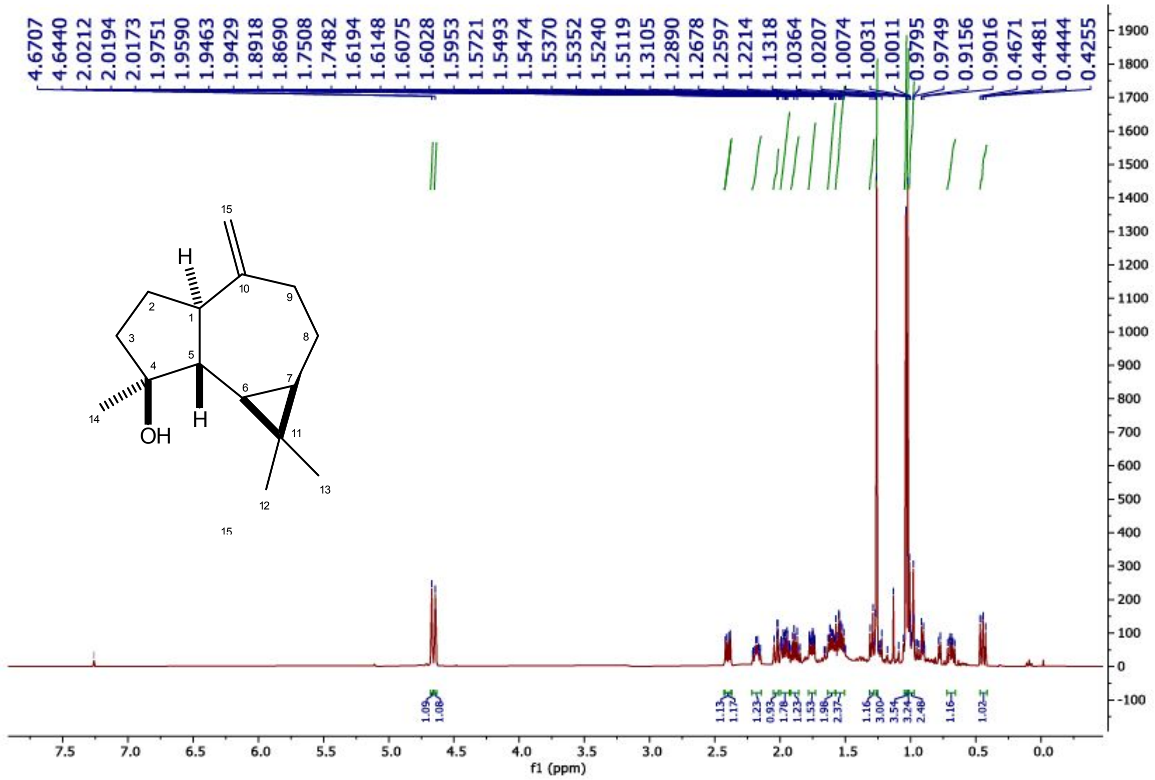

Figure S1 - ^{1}H NMR spectrum of spathulenol (δ, 600 MHz, $CDCl_3$).

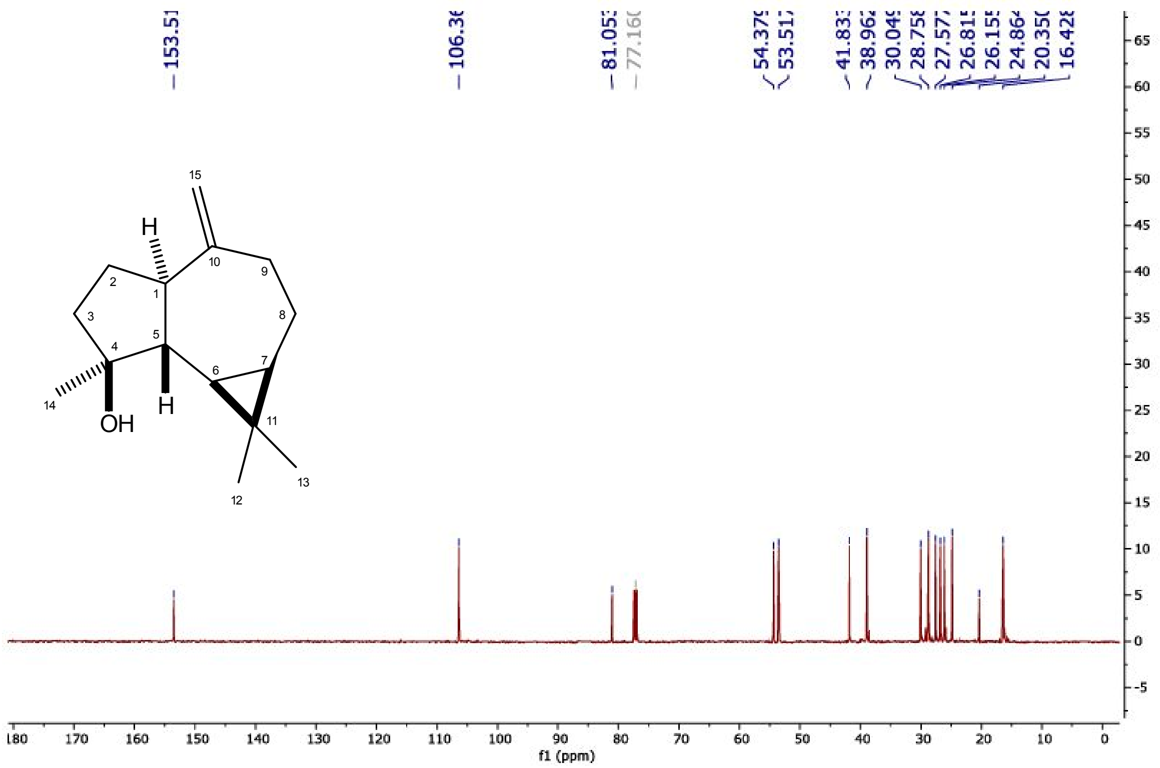

Figure S2 - ^{13}C NMR spectrum of spathulenol (δ, 150 MHz, $CDCl_3$).

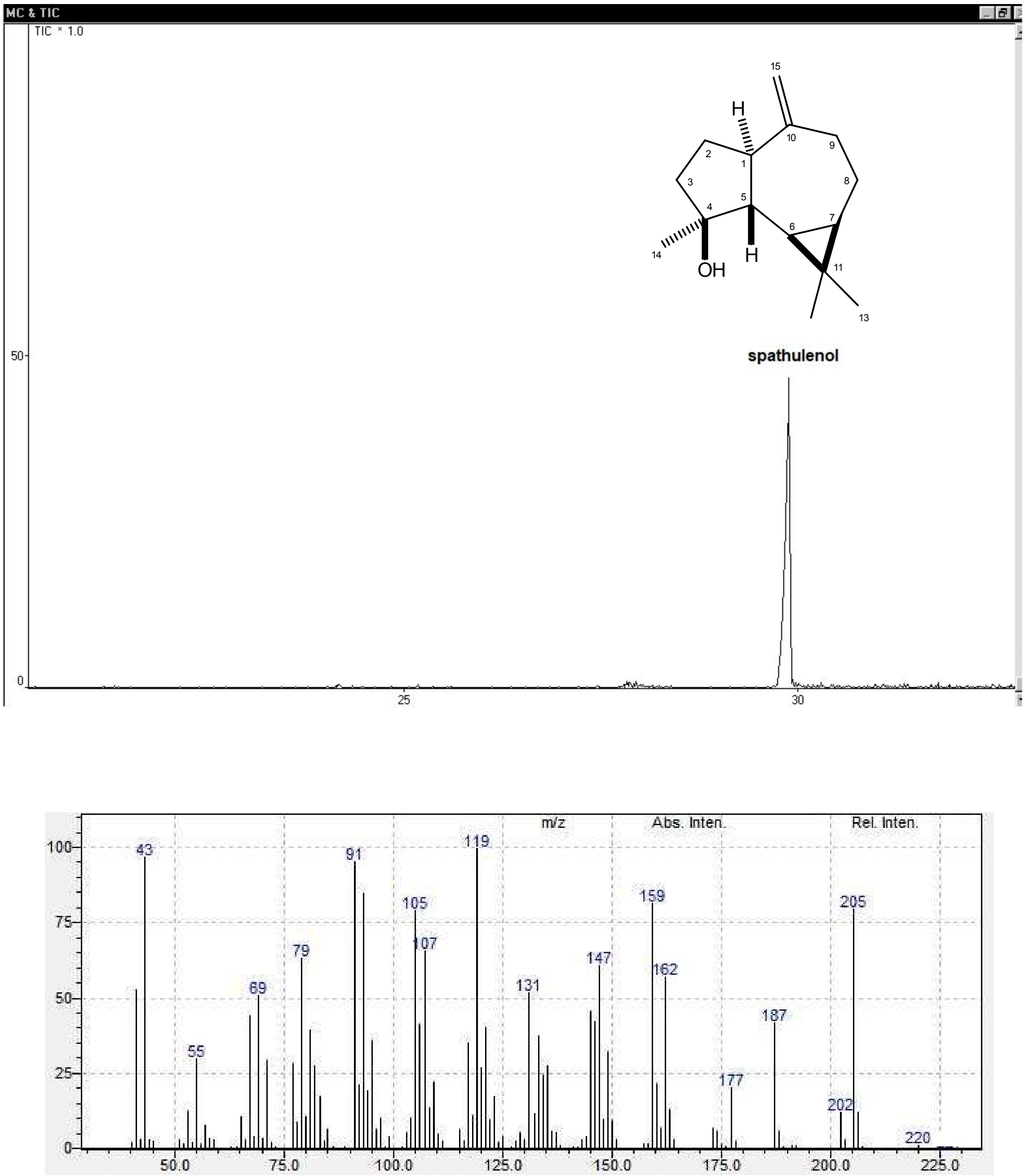

Figure S3 – GC/MS analysis (EI, 70 eV) of spathulenol.
